# Suppression of the RAC1/MLK3/p38 Signaling Pathway by β-Elemene Alleviates Sepsis-Associated Encephalopathy in Mice

**DOI:** 10.3389/fnins.2019.00358

**Published:** 2019-04-24

**Authors:** Cailong Pan, Yanna Si, Qinghai Meng, Ling Jing, Lu Chen, Yong Zhang, Hongguang Bao

**Affiliations:** ^1^Department of Anesthesiology, Nanjing First Hospital, Nanjing Medical University, Nanjing, China; ^2^School of Pharmacy, Nanjing University of Chinese Medicine, Nanjing, China; ^3^Jiangsu Key Laboratory of Neurodegeneration, Department of Pharmacology, Nanjing Medical University, Nanjing, China

**Keywords:** β-elemene, sepsis-associated encephalopathy, RAC1, MLK3, p38MAPK, microglia

## Abstract

It is still difficult to treat sepsis-associated encephalopathy (SAE) which is a diffuse brain dysfunction caused by sepsis, with excessive activation of microglia as one of the main mechanisms. Ras-related C3 botulinum toxin substrate 1 (RAC1) is proven to be a key molecule in the inflammatory signaling network. By using microglial cell line BV-2 and a mouse model of cecal ligation puncture (CLP), we herein evaluated the effects of β-elemene, an extract of *Curcuma zedoaria Rosc.*, on RAC1 signaling in microglia. β-Elemene decreased the expressions of pro-inflammatory cytokines [tumor necrosis factor-α (TNF-α), interleukin-1β (IL-1β), and IL-6] and attenuated translocation of nuclear factor-κB (NF-κB) p65 from the cytosol to the nucleus in BV-2 cells after lipopolysaccharide (LPS) treatment. It also inhibited the activation of RAC1, mixed-lineage protein kinase 3 (MLK3) and p38 mitogen-activated protein kinase (MAPK). The phosphorylation of the RAC1 Ser71 site was increased by β-elemene. Moreover, the learning and memory abilities of CLP mice in the water maze test and fear conditioning test were improved after β-elemene treatment. It reduced the expression of the microglial marker IBA1, significantly increased RAC1 Ser71 phosphorylation, and suppressed the RAC1/MLK3/p38 signaling activation and inflammatory response in the hippocampus. In conclusion, β-elemene effectively alleviated SAE in mice and inhibited the RAC1/MLK3/p38 signaling pathway in microglia, and might be an eligible potential candidate for SAE treatment.

## Introduction

Sepsis-associated encephalopathy (SAE) is a diffuse brain dysfunction caused by sepsis. Sonneville et al. (2017) recently reported that 53% of septic patients had SAE in intensive care unit (ICU), and SAE was associated with higher mortality rate, more consumption of ICU resources and longer hospital stay. In clinical practice, SAE is primarily manifested as changes of mental status, especially those of awareness/consciousness and cognition (Chaudhry and Duggal, 2014). Rodent models of sepsis suffer from long-term memory impairment (Barichello et al., 2005; Semmler et al., 2007; Chavan et al., 2012), and long-term potentiation deficiency has been found in the hippocampal sections prepared from mice receiving cecal ligation puncture (CLP) by electrophysiological techniques (Imamura et al., 2011). Besides, increase of Aβ and synaptophysin in the hippocampus of CLP rats aggravates cognitive deficits (Schwalm et al., 2014). Collectively, the hippocampus plays important roles in sepsis-induced learning and memory deficits. However, the mechanism of SAE is still unclear, and the treatment outcomes remain unsatisfactory, thereby urgently requiring new therapies.

It is well-documented that neuroinflammation played a key role in the onset and progression of SAE (Semmler et al., 2008; Piva et al., 2015). Microglia mainly regulate inflammatory response in the brain, the excessive activation of which leads to central nervous system damage upon SAE (Michels et al., 2014). P38 mitogen-activated protein kinase (MAPK), as a member of the MAPK family, is involved in the production of inflammatory mediators and the regulation of cytoskeletal stability (Cuenda and Rousseau, 2007). By intraperitoneally injecting lipopolysaccharide (LPS) into C57 mice, Kelly et al. (2003) activated p38 and inhibited LTP in the hippocampus, which were reversed by a p38 inhibitor SB203580. P38 MAPK has also been reported to phosphorylate various transcription factors, triggering the upregulation of inflammatory genes in the case of SAE (Jacob et al., 2011). Additionally, mixed lineage kinase 3 (MLK3) is a MAPK kinase kinase (MKKK) that mediates the phosphorylation of p38 MAPK by binding ras-related C3 botulinum toxin substrate 1 (RAC1), a GTP-binding proteins (Gallo and Johnson, 2002). RAC1 dominantly participates in cellular physiological activities such as cell adhesion, proliferation and cytoskeleton regulation. Over-activated RAC1 is involved in pathological processes such as oxidative stress and inflammatory response upon sepsis (Sanlioglu et al., 2001). Additionally, RAC1 markedly affects microglial-mediated neuroinflammation and neurotoxicity (D’Ambrosi et al., 2014). After RAC1 changes from an inactive GDP-binding state to an active GTP-binding state, downstream signaling is activated. GTP binding of RAC1 is reduced through phosphorylation at Ser71 site (Kwon et al., 2000), which then decreases the binding of RAC1 to MLK3 and negatively regulates its downstream MAPK signaling pathway in the rat hippocampus, finally mitigating cerebral ischemic injury (Zhang et al., 2006). Therefore, enhancing RAC1 phosphorylation may suppress microglial activation and neuroinflammation, as a feasible SAE therapy.

*Curcuma zedoaria Rosc.* has been widely used as an anti-inflammatory, analgesic or antitumor agent in clinical practice (Lobo et al., 2009). β-Elemene, an extract of *Curcuma zedoaria Rosc.*, has well-documented broad-spectrum anticancer effects (Li et al., 2010). Yao et al. (2008) reported that β-elemene exerted antitumor effects on glioblastoma cells depending on p38 MAPK. Although β-elemene also has evident therapeutic effects on non-neoplastic diseases such as inflammation and atherosclerosis (Zhang et al., 2010; Liu et al., 2015), but the underlying mechanisms remain elusive.

We herein hypothesized that β-elemene relieved CLP-induced cognitive dysfunction by suppressing the RAC1/MLK3/p38 signal pathway to inhibit microglial activation. The findings provide new insights into the clinical effects of β-elemene on SAE and the mechanism.

## Materials and Methods

### Reagents

β-Elemene (purity: 99%) was purchased from Shanghai Gaolang Chemical Technology Co., Ltd. (Shanghai, China). Antibodies against IBA1, p-RAC1 (S71), RAC1, MLK3, p-MLK3 (T211 + S281), p65, p-p65 (S536), p38 and p-p38 (T180 + Y182) were bought from Abcam (CA, United States). Enhanced chemiluminescence reagents was obtained from PerkinElmer (Waltham, MA, United States). Secondary antibodies were provided by Chemicon (Temecula, CA, United States). PrimeScript^TM^ II 1st Strand cDNA Synthesis Kit was purchased from Takara Biomedical Technology (Beijing) Co., Ltd. (Beijing, China). Antibody against glyceraldehyde-3-phosphate dehydrogenase (GAPDH) and other reagents were bought from Sigma-Aldrich (St. Louis, MO, United States).

### CLP Mouse Model

Male C57BL/6 mice aged 6–8 weeks were used to establish an SAE model by CLP. After anesthesia, the mice were ligated with a sterile 4-gauge wire to the distal end of the cecum (approximately 1.5 cm). A sterile 22-gauge needle was used to puncture the center of the occluded cecum, that was pushed back into the abdominal cavity and sutured. For the sham group, the abdominal cavity was opened without ligation or perforation. Afterward, 1 ml of prewarmed (37°C) normal saline was intraperitoneally injected for rehydration. The mortality rate was ≤20%. β-Elemene was dissolved in vehicle (0.1% DMSO) and the mice were intraperitoneally injected with different concentrations of β-elemene (10, 20, 40 mg/kg, once a day) for 7 days after CLP. β-Elemene was first injected 6 h after CLP.

### Sample Collection

Two hours after the last injection of β-elemene the animals were sacrificed. Samples were collected and stored in two ways: (Sonneville et al., 2017) The intact brain of the mice was fixed in 4% paraformaldehyde at 4°C for over 24 h. These samples were used for HE staining, immunohistochemical assay and immunofluorescence assay (Chaudhry and Duggal, 2014). The hippocampus tissue was frozen in liquid nitrogen for Western blot and RT-PCR.

### Morris Water Maze Test

From the 7th day after surgery, Morris water maze training was performed in a 1.25 m-diameter circular water pool that was filled with 25°C water to a depth of 30 cm. A transparent 10 cm-diameter columnar escape platform was placed 1 cm below the water surface. The time that a mouse entered water to climb the platform was defined as the latency time. If the mouse did not find the platform within 60 s, it was guided to the platform, placed there for 20 s and then removed. During training, the platform was placed in a quadrant of the pool, and the mouse was started from one of the 4 quadrants, facing the wall. The experiments were conducted at the same time every day for four consecutive days. Subsequently, the number and time of a mouse entering the target quadrant within 60 s were recorded (Liu et al., 2018).

### Fear Conditioning Test

From the 7th day after surgery, fear conditioning test was conducted by placing the mice in a 70% ethanol-wiped test box for conditioned reflex training by sound stimulation and foot electrical stimulation. The mice were placed in a chamber with an energizable bottom. After 2 min of adaption, a single-frequency sound signal (4.5 kHZ, 60 dB, 30 s) was given to the mice for 4 times (interval: 3 min). Then an electric shock (1 mA, 5 s) and an audible signal as the prompt were given 6 times (interval: 3 min; 18 min in total), and simultaneously ended. After 24 h, a context test to evaluate hippocampus-dependent memory was performed by placing the mice in the same chamber again without any stimulation. Cognitive deficits was assessed by measuring the amount of time the mouse demonstrated “freezing behavior,” which is defined as a completely immobile posture except for respiratory efforts (Wiltgen et al., 2005).

### Hematoxylin-Eosin (HE) Staining

The samples fixed in paraformaldehyde were dehydrated with 70, 80, 90, 95, and 100% ethanol solutions after being washed with flow water, transparentized with xylene, and paraffin-embedded into tissue blocks. The blocks were thereafter cut into 4 μm-thick sections, deparaffinized in xylene, rehydrated in 100, 95, 80, and 75% ethanol solutions, and rinsed with water for 5 min. Then the sections were stained with hematoxylin for 4 min, rinsed with water for 15 min, differentiated with 1% hydrochloric acid-ethanol solution for 5∼30 s until they became red, and rinsed with water for about 15 min. After being stained with eosin for 90 s, the sections were dehydrated with ethanol solutions at ascending concentrations, transparentized with xylene, covered with clean coverslips by using 1–2 drops of neutral resin, and quickly observed under a microscope.

### Western Blotting

Tissues or cells were placed in a pre-cooled glass grinder, added pre-cooled lysis buffer containing PMSF and phosphatase inhibitors, and thoroughly ground for lysis. After addition of an appropriate amount of 5× loading buffer, proteins were separated by SDS-PAGE and transferred to PVDF membranes. The membranes were blocked with 5% bovine serum albumin (BSA) at room temperature for 1 h and then incubated with specific primary antibodies against GAPDH(1:5000), IBA1 (1:1000), p-RAC1 (S71) (1:1000), RAC1 (1:1000), MLK3 (1:5000), p-MLK3 (T211 + S281) (1:1000), p65(1:1000), p-p65 (S536) (1:1000), p38(1:1000) and p-p38 (T180 + Y182) (1:1000) overnight at 4°C. The membranes were then developed by enhanced chemiluminescence reagents with secondary antibodies. The data were analyzed with the Molecular Imager (Gel DocTM XR, 170–8170, United States) and the associated software Quantity One-4.6.5 software (Bio-Rad Laboratories, United States).

### Immunohistochemical Staining

The tissue was taken out, and fixed in a paraformaldehyde solution and prepared into paraffin blocks. The blocks thereafter cut into 4 μm-thick sections, deparaffinized and rehydrated. Endogenous peroxidase in the tissue sections was inactivated with 3% H_2_O_2_. After being blocked by goat serum, the sections were incubated with primary antibodies overnight at 4°C and then with secondary antibodies for 1 h at 37°C, stained with DAB color development solution, counterstained with hematoxylin for 4 min, and observed under the microscope.

### RAC1 Assay

Rac activation assays were performed according to the manufacturer’s protocol of RAC1 pull-down activation assay biochem kit (Cytoskeleton, United States).

### RT-PCR

Total RNA was extracted by using Trizol reagent according to the manufacturer’s instructions and reverse-transcribed into cDNA. PCR experiments were then conducted with ABI7500 system (Life Technologies, United States) by TB Green^TM^ Premix Ex Taq^TM^ (Tli RNaseH Plus) and ROX plus kit [Takara Biomedical Technology (Beijing) Co., Ltd., China]. GAPDH was used as a housekeeping gene. The mRNA levels were calculated by using the ^ΔΔ^CT method. The sense and antisense primers (TNF-α, IL-1β, IL-6, and GAPDH) as follows were purchased from Sangon Biotech (Shanghai) Co., Ltd. (China). TNF-α: 5′-TACTGAACTTCGGGGTGATTGGTCC-3′ and 5′-CAGCCTTGTCCCTTGAAGAGAAC-3′. IL-1β: 5′-GCACTACAGGCTCCGAGATGAAC-3′ and 5′-TTGTCGTTGCTTGGTTCTCCTTGT-3′. IL-6: 5′-CCGGAGAGGAGACTTCACAG-3′ and 5′-GGAAATTGGGGTAGGAAGGA-3′. GAPDH: 5′-ACCACAGTCCATGCCATCAC-3′ and 5′-CACCACCCTGTTGCTGTAGCC-3′.

### Cell Culture

BV-2 cells were cultured in DMEM containing 10% fetal bovine serum, 100 U/mL penicillin, and 100 mg/mL streptomycin in a 37°C incubator containing 5% CO_2_. β-Elemene was dissolved in 0.1% DMSO, and the cells were treated with different concentrations of β-elemene (1, 5, 25 μM) for 24 h.

### Nuclear Factor-κB (NF-κB) P65 Nuclear Translocation Assay

BV-2 cells were plated in culture dishes with chamber slides inside, treated with LPS (1 μg/ml) for 12 h with or without β-elemene (25 μM), fixed by 4% paraformaldehyde for 30 min, blocked with 1% BSA, and incubated with primary antibodies overnight at 4°C and with secondary antibodies for 1 h at room temperature. After being stained with 1 μg/mL DAPI for 1 min, the cells were observed and photographed using a fluorescence microscope.

### Statistical Analysis

Statistical analysis was carried out using ANOVA, with Bonferroni posttest as the post hoc test. The results were expressed as mean ± standard deviation. *P* < 0.05 was considered statistically significant. All analyses were performed with GraphPad Prism Version 5.01 (GraphPad Software Inc., San Diego, CA, United States).

## Results

### β-Elemene Suppressed LPS-Induced Inflammation in BV-2 Cells

**FIGURE 1:**
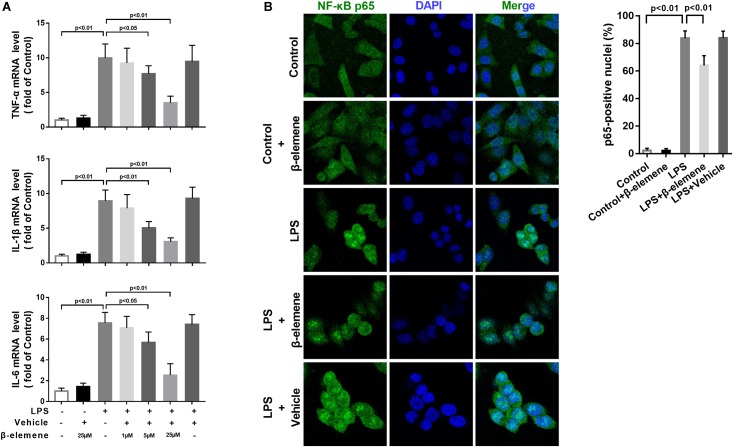
β-Elemene suppressed LPS-induced inflammation in BV-2 cells. **(A)** β-Elemene suppressed LPS-induced upregulation of mRNA expressions of TNF-α [F(6, 21) = 23], IL-1β [F(6, 21 = 26.4)], and IL-6 [F(6, 21 = 27.68)]. GAPDH was used as a loading control. **(B)** β-Elemene reduced the NF-κB translocation from the cytosol to the nucleus. F(4, 15) = 244.6. *n* = 4. Two-way or one-way ANOVA revealed a significant difference at *p* < 0.05.

To investigate the effect of β-elemene on LPS-induced microglial activation *in vitro*, we used a mouse microglial cell line BV-2, an alternative model system for primary microglia cultures or animal experiments examining brain inflammation (Henn et al., 2009). LPS treatment significantly upregulated the mRNA expressions of pro-inflammatory cytokines TNF-α, IL-1β, and IL-6, which were significantly reduced by β-elemene in a dose-dependent manner (Figure 1A). Hence, 25 μM of β-elemene was selected for subsequent experiments *in vitro*. Besides, β-elemene alone (25 μM) hardly affected the mRNA expressions of pro-inflammatory cytokines. In addition, LPS treatment significantly facilitated the translocation of p65 NF-κB from the cytoplasm to the nucleus, which was attenuated by β-elemene (Figure 1B).

### β-Elemene Inhibited the Activation of RAC1, MLK3, and p38 in BV-2 Cells

**FIGURE 2:**
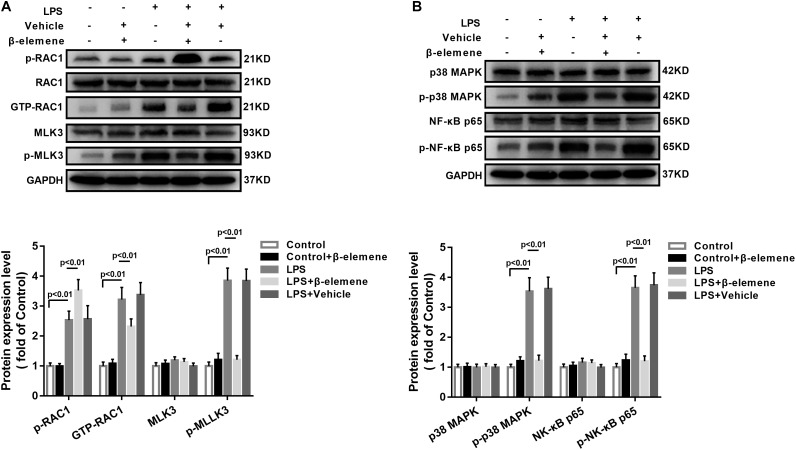
β-Elemene enhanced phosphorylation of RAC1 and weakened activation of RAC1, MLK3, and p38 in BV-2 cells. **(A)** β-Elemene enhanced RAC1 phosphorylation [F(4, 15) = 42.7] and weakened GTP-RAC1 production [F(4, 15) = 40.61] and MLK3 phosphorylation [F(4, 15) = 79.78]. Phosphorylation of RAC1 was normalized to that of RAC1. Other protein expressions were normalized to that of GAPDH. **(B)** β-Elemene significantly reduced the phosphorylation of p38 [F(4, 15) = 63.23] and p65 [F(4, 15) = 69.23] normalized to that of GAPDH. *n* = 4. Two-way ANOVA revealed a significant difference at *p* < 0.05.

Then we tested the activation of the RAC1/MLK3/p38 signaling pathway in BV-2 cells. The GTP-RAC1 was significantly increased by LPS induction, which was attenuated by β-elemene treatment (Figure 2A). The phosphorylation of MLK3 in BV-2 cells was significantly stronger than that of the control group, which was also weakened by β-elemene treatment (Figure 2A). Moreover, Western blotting exhibited that β-elemene significantly reduced the phosphorylation of p38 (Figure 2B). Interestingly, it significantly enhanced the phosphorylation of RAC1 Ser71 (Figure 2A). Taken together, the phosphorylated RAC1 Ser71 site was is involved in the regulation of RAC1 activity by β-elemene.

### β-Elemene Improved Learning and Memory in Septic Mice Induced by CLP

**FIGURE 3:**
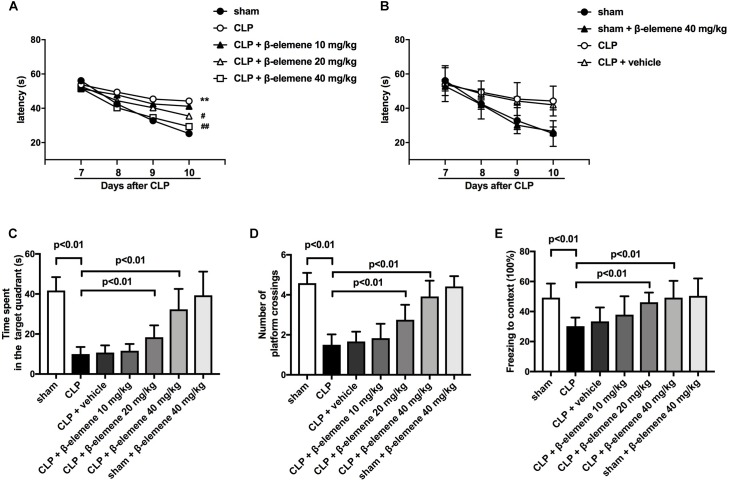
β-Elemene improved learning and memory in septic mice induced by CLP. **(A)** β-Elemene significantly shortened CLP-induced prolongation of CLP-induced latency time. F(6, 308) = 11. **(B)** β-Elemene did not affect the latency time of the sham group and vehicle did not influence that of CLP mice. F(6,308) = 11. **(C)** β-Elemene extended the time spent in the target quadrant. F(6, 77) = 45.56. **(D)** β-Elemene significantly increased the number of platform crossings. F(6, 77) = 54.78. **(E)** β-Elemene prolonged the freezing time in the context test. F(6, 77) = 8.943. *n* = 12. Two-way or one-way ANOVA revealed a significant difference at ^∗^*p* < 0.05 and ^∗∗^*p* < 0.01 versus sham group; and ^#^*p* < 0.05 and ^##^*p* < 0.01 versus CLP group.

We then evaluated the effects of β-elemene on the learning and memory of SAE mice. The water maze training showed that latency time was significantly prolonged after CLP compared to that of the sham group (Figure 3A), which was significantly shortened by β-elemene dose-dependently. There was no significant difference in the latency time between the β-elemene group and the sham group, or between the CLP group and the CLP + vehicle group (Figure 3B). After training, the platform was removed, and the time spent in the target quadrant and the number of platform crossings were detected. The time spent by CLP mice in the target quadrant was significantly shorter than that of the sham group, which, however, was significantly extended by β-elemene (Figure 3C). Moreover, the number of CLP mice crossing the platform was significantly reduced compared to that of the sham group, whereas β-elemene significantly increased the number in a dose-dependent manner (Figure 3D). Additionally, the fear conditioning test showed that the freezing time of the CLP group mice was significantly shorter than that of the sham group, which was prolonged by β-elemene treatment (Figure 3E). Hence, 40 mg/kg of β-elemene was selected for subsequent experiments. Notably, β-elemene administration did not affect the learning or memory of the sham group, and the placebo effect of vehicle on CLP mice was negligible.

### β-Elemene Reduced Microglial Marker IBA1 Expression in the Hippocampus of CLP Mice

**FIGURE 4:**
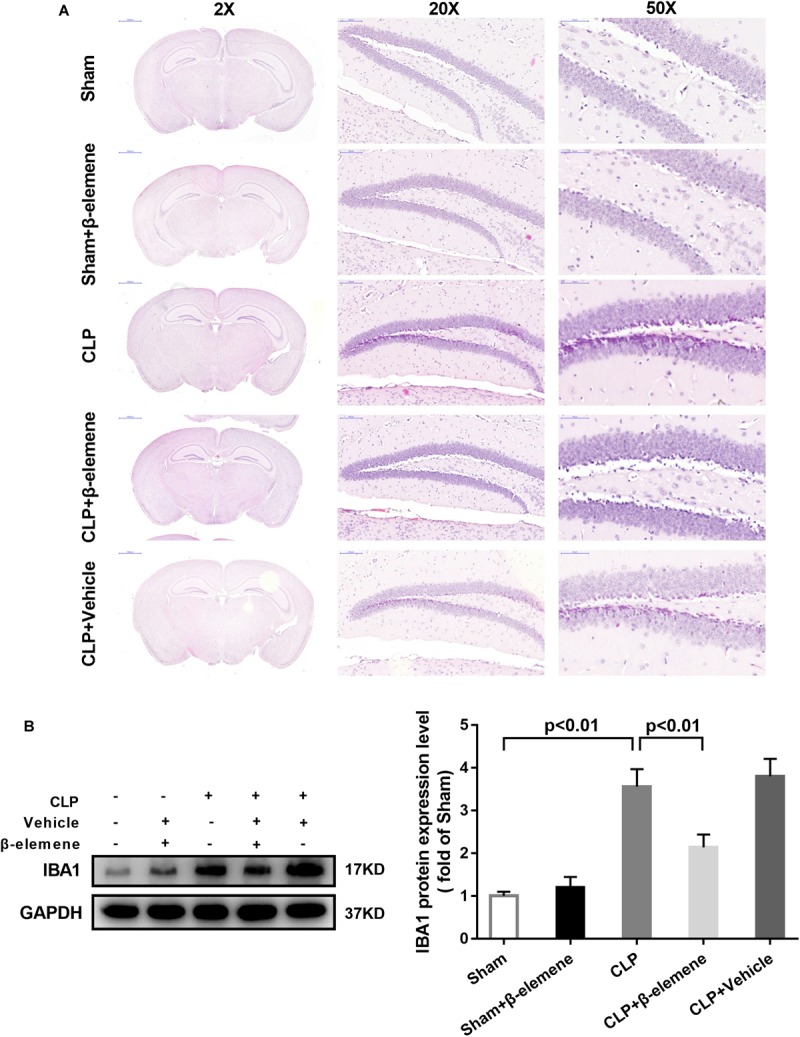
β-Elemene reduced the expression of the microglial marker IBA1 in the hippocampus of CLP mice. **(A)** Images of HE-stained brains from sham and β-elemene groups. Magnification: 20, 200, or 500×. **(B)** β-Elemene reduced IBA1 expression in the hippocampus normalized to that of GAPDH. F(4, 15) = 46.08. *n* = 4. One-way ANOVA revealed a significant difference at *p* < 0.05.

**FIGURE 5:**
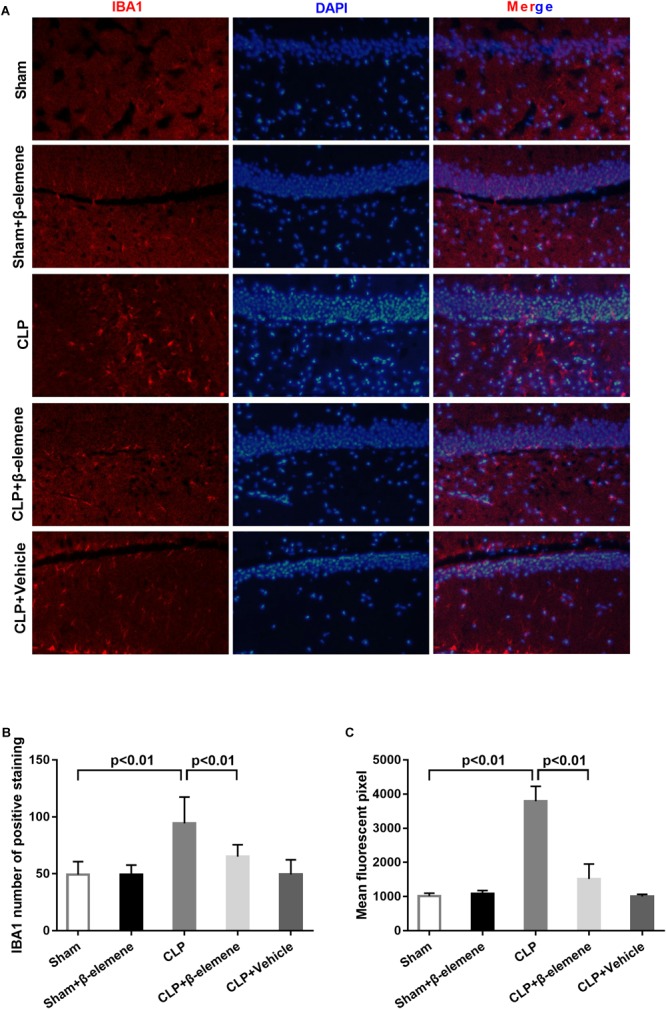
β-Elemene reduced microglial activation in the hippocampus of CLP mice. **(A)** Immunofluorescence staining of IBA1 in the brains of different groups. Magnification: 200×. **(B)** IBA1 numbers of positively stained hippocampal dentate gyri in different groups. F(4, 15) = 53.87. **(C)** Mean fluorescent pixels of IBA1 in the hippocampal dentate gyri of different groups. F(4, 15) = 8.416. *n* = 4. One-way ANOVA revealed a significant difference at *p* < 0.05.

We further examined the hippocampal changes of CLP mice. HE staining showed that no abnormalities in brain cells were observed in the sham group. In contrast, largest amount of nuclear pyknosis were observed in the CLP groups. The dentate gyrus region changed most significantly. Moreover, mice given β-elemene showed significantly fewer abnormal cells compared to the CLP group (Figure 4A). Furthermore, Western blotting and immunofluorescence assay showed that the hippocampal expression of microglial marker IBA1 was significantly raised in CLP mice compared with that of the sham group, which was reduced by β-elemene treatment (Figure 4B, 5).

### β-Elemene Increased RAC1 Phosphorylation and Suppressed Its Activation in the Hippocampus

**FIGURE 6:**
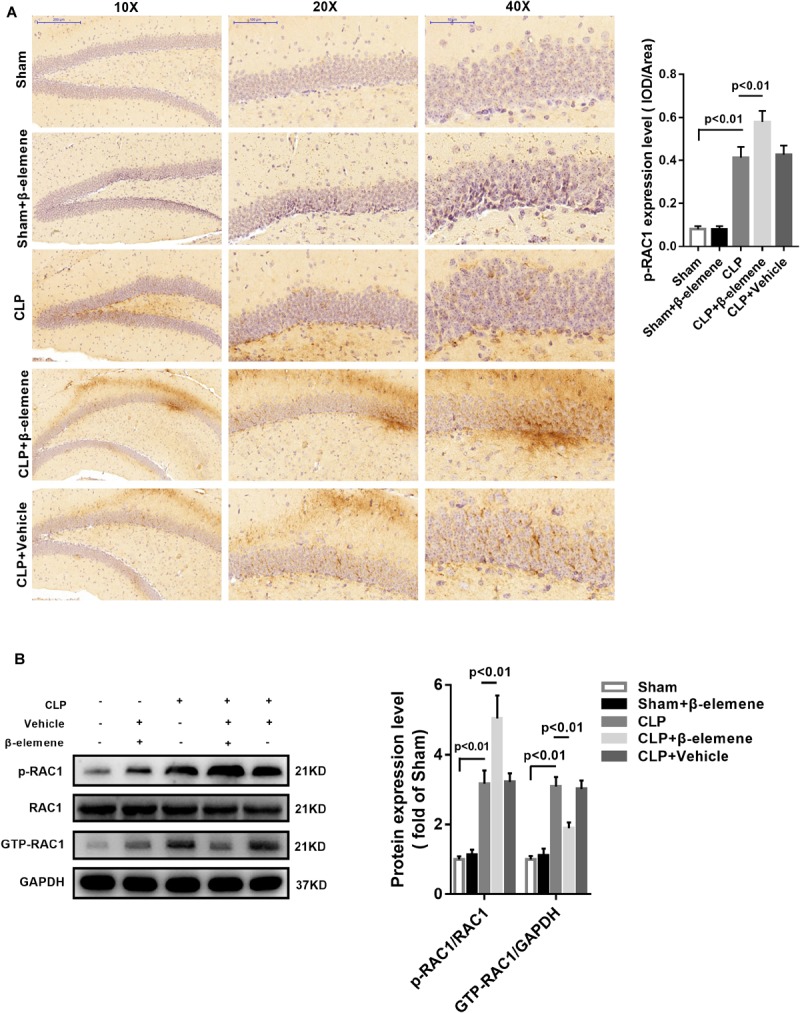
β-Elemene increased the RAC1 phosphorylation in the mouse hippocampus. **(A)** Immunohistochemical staining showed that β-elemene significantly facilitated RAC1 phosphorylation. F(4, 15) = 102. **(B)** Western blotting exhibited that β-elemene significantly increased RAC1 phosphorylation [F(4, 15) = 72.76] and decreased GTP-RAC1 [F(4, 15) = 63.42] detected by western blotting in the hippocampus. Phosphorylation of RAC1 was normalized to that of RAC1. GTP-RAC1 expression was normalized to that of GAPDH. *n* = 4. One-way ANOVA revealed a significant difference at *p* < 0.05.

Immunohistochemical assay exhibited that CLP itself induced moderate phosphorylation of RAC1, whereas β-elemene administration significantly augmented RAC1 Ser71 phosphorylation (Figure 6A). Western blotting results further confirmed that β-elemene significantly increased RAC1 phosphorylation and decreased GTP-RAC1 in the mouse hippocampus (Figure 6B).

### β-Elemene Reduced MLK3 Phosphorylation in the Hippocampus

**FIGURE 7:**
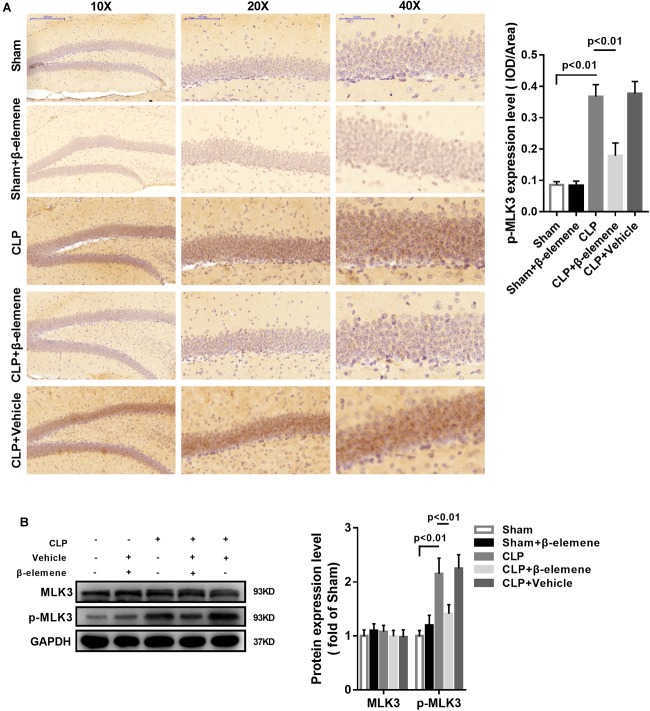
β-Elemene reduced MLK3 phosphorylation in the hippocampus of CLP mice. **(A)** Immunohistochemical staining exhibited that β-elemene significantly attenuated MLK3 phosphorylation. F(4, 15) = 64.04. **(B)** Western blotting presented that β-elemene significantly reduced MLK3 phosphorylation [F(4, 15) = 24.27] normalized to that of GAPDH. *n* = 4. One-way ANOVA revealed a significant difference at *p* < 0.05.

Immunohistochemical assay showed that the phosphorylation of MLK3 in the hippocampus of CLP mice was significantly enhanced compared with that of the sham group, which was attenuated by β-elemene treatment (Figure 7A). In addition, β-elemene significantly weakened the phosphorylation of MLK3 in the hippocampus, as suggested by Western blotting (Figure 7B).

### β-Elemene Reduced p38 MAPK Phosphorylation, and Expressions of Pro-inflammatory Cytokines in the Hippocampus of CLP Mice

**FIGURE 8:**
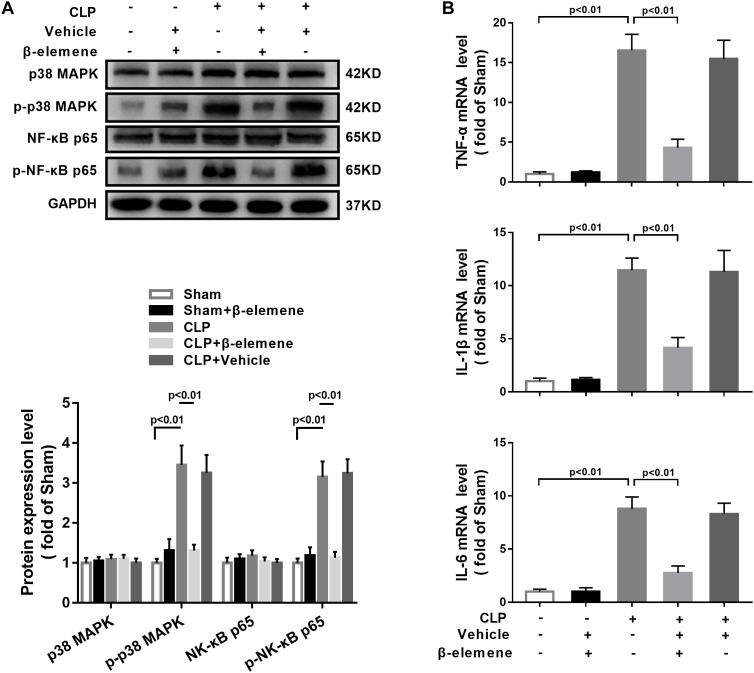
β-Elemene reduced p38 MAPK phosphorylation and expressions of pro-inflammatory cytokines in the hippocampus of CLP mice. **(A)** Western blotting detected that β-elemene significantly weakened the phosphorylation of p38 [F(4, 15) = 44.95] and p65 [F(4, 15) = 58.7] normalized to that of GAPDH. **(B)** RT-PCR detected that β-elemene significantly reduced the mRNA expressions of TNF-α [F(4, 15) = 83.98], IL-1β [F(4, 15) = 64.01] and IL-6 [F(4, 15) = 73.18]. GAPDH was used as a loading control. *n* = 4. Two-way ANOVA revealed a significant difference at *p* < 0.05.

We further assessed the effect of β-elemene on CLP-induced upregulation of p38 MAPK and NF-κB p65 phosphorylation. Western blotting presented that β-elemene significantly reduced the phosphorylations of both p38 MAPK and NF-κB p65 (Figure 8A). Furthermore, RT-PCR indicated that CLP mice had higher mRNA expression levels of pro-inflammatory molecules (TNF-α, IL-1β, and IL-6) than those of the sham group, whereas β-elemene effectively reduced them (Figure 8B).

## Discussion

Herein, β-elemene suppressed the activation of RAC1, MLK3, p38 MAPK, and NF-kB p65, as well as significantly decreased the expressions of pro-inflammatory cytokines in BV-2 cells and the hippocampus of mice. Moreover, it mitigated the learning/memory deficit in septic mice induced by CLP. Compared with the CLP group, β-elemene lowered the expression of the microglial marker IBA1 and increased the phosphorylation of RAC1 in the hippocampus. Taken together, β-elemene may be able to combat SAE via the RAC1/MLK3/p38 signaling pathway, as an attractive treatment candidate.

Accumulating evidence has proven that RAC1, as a key molecule in the inflammatory signaling network, controlled the progression of inflammation. Kuijk et al. (2008) found that RAC1 was responsible for the release of pro-inflammatory cytokines, which, when inhibited, significantly decreased IL-1β. Zhang et al. (2011) reported that RAC1 was activated during LPS stimulation, thereby inducing TNF-α expression. Furthermore, Yao et al. (2011) used RAC1 inhibitor NSC23766 to significantly attenuate pro-inflammatory cytokines release, inflammatory cell infiltration, neutrophil migration and endothelial cell permeability in a mouse model of LPS-induced pulmonary inflammation. Similarly, Hwaiz et al. (2013) found that RAC1 was an effective target for protecting against lung injury during sepsis. NSC23766 markedly reduced CLP-triggered neutrophil infiltration, edema formation, and lung damage. Qin et al. (2015) also thought that RAC1 may be an effective therapeutic target for inflammation and sepsis. In this study, we found that inhibiting RAC1 activity also had protective effects on SAE. Meanwhile, the phosphorylation of RAC1 Ser71 site may be involved in the reduction of GTP-RAC1 by β-elemene. Although RAC1 Ser-71-phosphorylation weakened GTP binding without affecting the GTPase activity, phosphorylation predominantly modulated the subcellular localization of the GTPase, thereby negatively affecting its activity (Kwon et al., 2000; Riento et al., 2005; Wiltgen et al., 2005; Zhang et al., 2006, 2010, 2011; Kuijk et al., 2008; Yao et al., 2008, 2011; Henn et al., 2009; Lobo et al., 2009; Schoentaube et al., 2009; Li et al., 2010; Schwarz et al., 2012; Hwaiz et al., 2013; Liu et al., 2015, 2018; Qin et al., 2015). In addition, RAC1 Ser71 was moderately phosphorylated in the hippocampus of CLP mice (Figure 6A,B). RAC1 Ser71 phosphorylation in the CLP mice may be implicated as a subsequent protective reaction by the cell itself, but not a damage factor in the pathological process of SAE. In this study, for the first time, we found that β-elemene suppressed the inflammatory responses in CLP mice and LPS-exposed BV-2 cells likely by augmenting RAC1 Ser71 phosphorylation.

RAC1 has been reported to play important roles in cell movement, tumor angiogenesis and invasion/metastasis (Bid et al., 2013). Since RAC1 inhibitor NSC23766 is capable of combating breast cancer and lung cancer (Zuo et al., 2006; Hernandez et al., 2010; Yoshida et al., 2010; Chen et al., 2011), the antitumoral effects of β-elemene may also involve the same pathway. In addition, β-elemene can potently inhibit pro-inflammatory cytokines and protect against endotoxin-induced inflammation (Fang et al., 2018). Similarly, we found that β-elemene reduced the expressions of pro-inflammatory factors in the mouse model of SAE (Figure 6B). Jackson et al. found that the activated form of RAC1 promoted the autophosphorylation and substrate phosphorylation of MLK3, as an upstream activator of the p38 pathway (Jackson et al., 2016). In this study, β-elemene reduced MLK3 and p38 phosphorylation in SAE mice (Figure 5, 6A). Notably, injectable emulsion of β-elemene has been developed and approved by the CFDA for clinical cancer therapy (Zhai et al., 2018). Therefore, it is a novel choice for SAE treatment.

However, this study still has some limitations. First, the possibility that β-elemene inhibited neuroinflammation by affecting intraabdominal inflammation cannot be ruled out, because it was intraperitoneally administered. Given that elemene can pass through the blood–brain barrier (Wu et al., 2009), β-elemene may directly suppress microglia-mediated neuroinflammation, playing a major role in relieving the cognitive impairment of CLP mice. Second, this study was only preliminarily explored the relationship between β-elemene and RAC1, so the regulatory mechanisms still need further in-depth studies. In summary, β-elemene alleviated SAE in mice by suppressing the RAC1/MLK3/p38 signaling pathway. Therefore, β-elemene may be a potential candidate for treating SAE.

## Ethics Statement

All subjects gave written informed consent in accordance with the Declaration of Helsinki. The protocol was approved by the Nanjing Medical University Animal Care and Use Committee.

## Author Contributions

CP designed the experiments, performed the experiments, analyzed the results, and drafted the manuscript. YS and QM carried out the behavioral measure. LJ carried out the cell cultures. LC and YZ carried out the western blotting analysis, immunohistochemical staining, and RT-PCR analysis. HB conceived of the study, participated in its design and coordination, and helped to draft the manuscript. All the authors read and approved the final manuscript.

## Conflict of Interest Statement

The authors declare that the research was conducted in the absence of any commercial or financial relationships that could be construed as a potential conflict of interest.
